# Inactivation of the *pgmA* Gene in *Streptococcus mutans* Significantly Decreases Biofilm-Associated Antimicrobial Tolerance

**DOI:** 10.3390/microorganisms7090310

**Published:** 2019-09-03

**Authors:** Martin Nilsson, Michael Givskov, Svante Twetman, Tim Tolker-Nielsen

**Affiliations:** 1 Costerton Biofilm Center, Department of Immunology and Microbiology, Faculty of Health and Medical Sciences, University of Copenhagen, DK-2200 Copenhagen, Denmark; 2 Singapore Centre for Environmental Life Sciences Engineering, Nanyang Technological University, Singapore 637551, Singapore; 3 Department of Odontology, Faculty of Health and Medical Sciences, University of Copenhagen, DK-2200 Copenhagen, Denmark

**Keywords:** *Streptococcus mutans*, *pgmA*, biofilm, antimicrobial tolerance

## Abstract

Screening of a *Streptococcus mutans* mutant library indicated that *pgmA* mutants displayed a reduced biofilm-associated tolerance toward gentamicin. The biofilms formed by the *S. mutans* *pgmA* mutant also displayed decreased tolerance towards linezolid and vancomycin compared to wild-type biofilms. On the contrary, the resistance of planktonic *S. mutans* *pgmA* cells to gentamycin, linezolid, and vancomycin was more similar to wild-type levels. Investigations of biofilms grown in microtiter trays and on submerged glass slides showed that *pgmA* mutants formed roughly the same amount of biofilm as the wild type, indicating that the reduced antimicrobial tolerance of these mutants is not due to diminished biofilm formation. The *pgmA* gene product is known to be involved in the synthesis of precursors for cell wall components such as teichoic acids and membrane glycolipids. Accordingly, the *S. mutans* *pgmA* mutant showed increased sensitivity to Congo Red, indicating that it has impaired cell wall integrity. A changed cell wall composition of the *S. mutans* *pgmA* mutant may play a role in the increased sensitivity of *S. mutans* *pgmA* biofilms toward antibiotics.

## 1. Introduction

*Streptococcus mutans* is a part of the complex microbiota of the oral cavity and can promote dental caries under some conditions [1]. The ability to form biofilm is an essential feature of *S. mutans* pathogenicity [2]. The biofilm that develops on the tooth surface, termed dental plaque, is one of the most well-studied multispecies biofilm-systems [3]. In the sucrose dependent adherence pathway, *S. mutans* expresses three glucosyltransferases and a fructosyltransferase, which act on sucrose and synthesize glucan- and fructan-polymers, respectively. The glucan-polymers promote adhesion and biofilm formation on teeth, whereas the fructan- polymers serve as storage compounds which can be used during periods of nutrient deprivation [4,5]. In addition, *S. mutans* can cause extra-oral infections such as infective endocarditis [6]. Through lesions in the oral cavity, *S. mutans* can reach the blood circulation [7]. These small bacteremia are normally rapidly cleared by the immune defense, but in some cases, e.g., if the host has damaged heart valves, *S. mutans* can colonize the heart valves and establish a biofilm [8,9,10]. 

Bacteria in biofilms can resist host immune responses and tolerate exposure to much higher doses of antibiotics than planktonic bacteria, and biofilms can therefore be the cause of persistent infections [11,12]. The mechanisms underlying the antibiotic tolerance of biofilms include restricted antibiotic penetration, the presence of bacteria in various physiological states, and the expression of specific genes which give antimicrobial tolerance [13,14]. However, more knowledge about the factors that are involved in biofilm-associated antimicrobial tolerance is needed for future development of medicine for efficient treatment of biofilm infections.

We recently constructed a *S. mutans* transposon mutant library and screened for mutants that display reduced biofilm-associated antimicrobial tolerance [15,16,17]. In these studies, we found that the teichoic acid-modifying Dlt system, as well as the oxidative stress response regulator SpxA1, play a role in antibiotic tolerance of *S. mutans* biofilms [16,17]. In the present study, we present evidence that the *pgmA* gene has a role in the antimicrobial tolerance displayed by *S. mutans* biofilms. The *pgmA* enzyme is involved in the synthesis of precursors for lipoteichoic acids, wall teichoic acids, membrane glycolipids, capsules and exopolysaccharides. The available evidence suggests that impaired cell wall integrity may render *S. mutans pgmA* biofilms susceptible to antibiotics.

## 2. Materials and Methods

### 2.1. Bacterial Strains and Growth Conditions

The bacterial strains used in this study are listed in Table 1. For routine cultivation, the *S. mutans* UA159 wild type and mutants were grown in Tryptone soy medium (TSB) or Todd-Hewitt medium (THB). *S. mutans* cultures were grown static in a 5% CO_2_ incubator at 37 °C unless otherwise stated. Tryptone soy agar (TSA) was used for plating and the plates were incubated anaerobically (10% H_2_, 10% CO_2_, and 80% N_2_) or in a 5% CO_2_ incubator at 37 °C. THB supplemented with 1% sucrose (THBS) was used to cultivate *S. mutans* biofilms. *Escherichia coli* strains were grown in Luria-Bertani medium at 37 °C. When appropriate, antibiotics were used for bacterial cultures at the following concentrations: For *S. mutans* strains, erythromycin at 10 µg/mL, spectinomycin at 1 mg/mL; for *E. coli* strains, kanamycin at 50 µg/mL.

### 2.2. Transformation of S. mutans

The transformation protocol is based on a previously published procedure [22]. An *S. mutans* overnight culture grown anaerobically in THB was diluted 1:20 in THB containing 5% heat inactivated horse serum (PAA). The cultures were incubated anaerobically at 37 °C. When they reached an OD_600_ between 0.15 and 0.25, competence-stimulating peptide (CSP) with the amino acid sequence of NH2-SGSLSTFFRLFNRSFTQALGK-COOH (purity >95%, TAG Copenhagen A/S) was added to a final concentration of 500 µg/mL together with DNA. Cultures were incubated for 90 min, concentrated by centrifugation, and spread on TSA plates containing erythromycin.

### 2.3. Screening of a S. mutans Transposon Library for Mutants Impaired in Biofilm-Associated Gentamicin Tolerance

Primary screenings were done similar to the procedure published by Thien-Fah Mah and coworkers [23]. Colony spots were plated directly from transposon library microtiter plate glycerol stocks onto erythromycin-containing TSA plates (14 cm diameter petri dishes) using a replicator with 3 mm pins. The plates were incubated at 37 °C anaerobically. Then, 96-well microtiter plates containing 100 µL THBS per well, were inoculated from the plates with colony spots using the 96-pin replicator and were incubated aerobically at 37 °C. After 24 h of incubation, the medium in each well was replaced with 125 µL THBS containing 37.5 µg/mL gentamicin (eight times lower than the minimal bactericidal concentration for biofilms (MBC-B) value of the wild type). The biofilms present on the wall of the wells were exposed to the gentamicin for 24 h, and subsequently incubated with 150 µL THB without any antibiotics (recovery medium) for an additional 24 h. To subsequently quantify bacterial viability, the 96-pin replicator was used to plate the cultures on TSA plates without antibiotics.

### 2.4. Determination of Minimum Bactericidal Concentration for Biofilm Cells (MBC-B) and Minimum Bactericidal Concentration for Planktonic Cells (MBC-P)

Determination of MBC-B values was done using overnight cultures that were diluted to an OD_600_ of 0.02 in THBS. Cultures arranged in triplicates were grown at 37 °C in 100 µL THBS in microtiter tray wells. The medium was then removed and biofilms on the wall of the wells were exposed to two-fold serial dilutions of antibiotics in THB that were added at 125 µL per well. The cultures were incubated for 24 h, the medium was discarded, and 150 µL recovery medium was added to the wells. After additional 24 h incubation, the viability of the cultures was analyzed by spotting small amounts of the cultures on TSA plates using a 96-pin replicator. The lowest concentration of antibiotic resulting in no viability in the spotted area was considered to be the MBC-B value.

The MBC-P values were determined using overnight cultures that were diluted to an OD_600_ of 0.02 in THB. Antibiotics in two-times serial dilutions were added to the bacteria in 150 μL microtiter tray cultures that subsequently were incubated for 24 h, followed by plating and evaluation of viability as described above.

### 2.5. Time-Kill Assay

Overnight cultures of the *S. mutans* wild type and *pgmA* mutant were diluted 50× in THBS, and were added to the wells of microtiter trays (100 µL per well), and grown for 24 h at 37 °C. The medium was subsequently replaced with THB or THB supplemented with 20 µg/mL gentamicin to challenge the biofilms on the walls of the wells with antibiotics. At 0, 1, 2, and 3 h, the biofilms were washed twice with 150 µL 0.9% NaCl and were then disrupted by sonication for 5 s (1% amplitude with a 600-Watt Ultrasonic processor). To determine CFUs, the cells from the disrupted biofilms were diluted in 0.9% NaCl in 10-fold steps, and 20 µL of each dilution was spotted on TSA agar plates. Colonies formed on the agar plates were counted after two days incubation. The time-kill assays were performed in triplicate. It was assured that the sonication step did not decrease the viability of the wild type and *pgmA* mutant bacteria (data not shown).

### 2.6. Identification of Interrupted Genes by Arbitrary Primed Polymerase Chain Reaction (PCR)

In order to identify the location of the mariner transposon insertions in the selected mutants, the arbitrary primed PCR protocol outlined by Li et al. was followed [24]. Briefly, in the first round of PCR, one of the arbitrary primers was paired with a primer that reads out from the erythromycin resistance gene. Approximately 100 ng of purified chromosomal DNA, obtained using Qiagen’s DNeasy^®^ Blood & Tissue kit, was used as template. In the second PCR, a part of the product from the first PCR was PCR amplified with an arb-3 primer and a nested *erm* primer. Purified second-round products were used for DNA sequencing performed by Macrogen by the use of the nested *erm* primer. The insertion sites were identified with BLASTN searches, against the annotated sequence of *S. mutans* UA159, using software provided by NCBI.

### 2.7. Construction of a pgmA Deletion Mutant

A *pgmA* (*Smu_1077*) deletion mutant was generated using PCR ligation mutagenesis [25]. In short, upstream and downstream fragments were PCR amplified using purified DNA of *S. mutans* UA159 as template and the primer pair 1077-P1/1077-P2 and 1077-P3/1077-P4. The upstream PCR products were digested with *Asc*I and the downstream PCR products were digested with *Fse*I. The upstream and downstream fragments were separately ligated with an amplified *Asc*I/*Fse*I cleaved *erm* cassette. The upstream-erm and the downstream-erm ligations were subsequently mixed and overlap extension PCR with the P1 and P4 primers was performed. The resulting PCR fragment was purified and transformed into *S. mutans* UA159 using erythromycin selection. Mutant candidates were screened for the presence of the correct gene deletion using the PCR method described previously [24]. The sequences of the PCR primers are listed in Table 1.

### 2.8. Construction of pgmA Complemented Strain

To complement the *pgmA* (*Smu_1077*) knockout mutant strain, a PCR fragment of *pgmA* comprising the entire open reading frame and promoter sequence was generated using primers 1077compF/1077compR. The fragments were digested with *Bam*HI and ligated into the corresponding site of the shuttle vector pDL277 [20]. The resulting plasmid, termed pDL277-pgmA, was transformed into the *pgmA* mutant strain, resulting in *S. mutans pgmA*/pDL277-pgmA. In addition, the vector control strain *S. mutans pgmA*/pDL277 was generated. The sequences of the PCR primers are listed in Table 1.

### 2.9. Crystal Violet Biofilm Assay

Biofilm formation in microtiter plates was quantified by crystal violet (CV) staining, essentially as described by O’Toole and Kolter [26]. An *S. mutans* overnight culture grown in THB was diluted to an OD_600_ of 0.02 in THBS, and 100 μL of the diluted culture was added to the wells of a 96-well microtiter plate, which was subsequently incubated at 37 °C for 24 h. The wells were aspirated and remaining planktonic bacteria were removed by addition and removal of 120 μL Milli-Q water (MQH_2_O). The biofilms were stained for 15 min with 120 μL 0.4% CV. CV quantification was done by washing the wells twice with 150 μL MQH_2_O, solubilizing the CV with 30% acetic acid for 30 min, and measuring the absorbance at 590 nm.

### 2.10. CLSM and Airyscan

UV sterilized cover glasses were used as substratum for biofilms. Overnight cultures of *S. mutans* strains were grown in THB, diluted 50-fold in fresh THBS, and poured into large petri dishes (80 mL per petri dish), each containing sterilized cover glasses. The cultures were grown for 24 h at 37 °C. Subsequently, the biofilms on the cover glasses were carefully rinsed with PBS and stained for 15 min with Syto 9 diluted 1000 times in PBS. The biofilms were washed in PBS and mounted, with the biofilm side down, on microscope slides with silicon-made wells containing PBS. Confocal laser scanning microscopy (CLSM) was performed on the cover classes using a Zeiss LSM710 microscope with a Plan-Apochromat 63×/1.4 oil DIC immersion lens. CLSM-captured images and COMSTAT software with a fixed threshold value of 15, were used to quantify biofilms [27]. Airyscan images were obtained with a Zeiss LSM880 with Airyscan and Plan-Apochromat 63×/1.4 oil DIC lens. An argon laser, at 488 nM, was used as an excitation source for the fluorescent dye both with the Zeiss LSM710 and Zeiss LSM880 microscope. Images were further processed using IMARIS (Bitplane, Zürich, Switzerland) software.

### 2.11. Congo Red Susceptibility Assay

A Congo Red susceptibility assay was performed essentially as described previously [28]. Overnight cultures of the *S. mutans* wild type and *pgmA* mutant were diluted in 0.9% NaCl with 10-fold steps and spotted on TSA agar and TSA agar supplemented with 0.2% (*wt*/*vol*) Congo Red. Plates were incubated at 37 °C for 48 h and subsequently imaged.

### 2.12. Statistics

Statistical analysis was done with the t-test (two-sample, assuming equal variances) using the Analysis ToolPak of Excel. Differences were considered to be significant when *P* < 0.05 was obtained.

## 3. Results

### 3.1. S. mutans pgmA Mutants Display Reduced Biofilm-Associated Antimicrobial Tolerance

A *S. mutans* mariner transposon mutant library containing approximately 5000 mutants [15] was screened for mutants that displayed reduced biofilm-associated gentamicin tolerance. Mutants that showed lowered biofilm tolerance were rescreened, and those mutants that repeatedly exhibited lowered gentamicin tolerance were selected for further analysis. Sequence analyses of the regions flanking the transposon inserts in the selected mutants showed that two mutants had the transposon inserted in the *Smu_1077* gene albeit at different positions.

*Smu_1077* is annotated in the *S. mutans* UA159 genome project to be a phosphoglucomutase gene. BLASTP searches with the annotated protein sequence of *Smu_1077*, which comprise 571 amino acids, showed 47% identity to the protein denoted α–phosphoglucomutase (α–PGM) in *Bacillus subtilis*. In addition, sequence analysis indicated that the *Smu_1077* gene product contains the characteristic functional regions of the α–PGM protein, including a metal binding region and active site residues. We therefore use the designation *pgmA* for the *Smu_1077* gene. α–phosphoglucomutase mediates the reversible reaction that converts glucose 6-phosphate to glucose 1-phosphate, which is a precursor of uridine diphosphate-glucose (UDP-glucose) [29]. In Gram-positive bacteria, UDP-glucose is a precursor in the synthesis of many cell wall components, e.g., lipoteichoic acids, wall teichoic acids, membrane glycolipids, capsules, and exopolysaccharides.

The gentamicin minimal bactericidal concentration for biofilm cells (MBC-B) value for the *S. mutans pgmA* transposon mutant was eight-fold lower than that of the wild type, whereas the gentamicin minimal bactericidal concentration for planktonic cells (MBC-P) value for the *pgmA* mutant was around four-fold lower than wild type levels (Table 2). To confirm that the disruption of the *pgmA* gene by the transposon was responsible for the observed phenotypes, we constructed a defined *pgmA* knockout mutant and determined the MBC-B and MBC-P values for this mutant with respect to gentamicin. The biofilm formed by the *pgmA* knockout mutant showed a similar reduction in gentamicin tolerance as the transposon mutant biofilm, and the MBC-P values were also similar (Table 2). We also constructed a *S. mutans* strain where the *pgmA* mutation is complemented with a plasmid-borne *pgmA* gene. The biofilm-associated tolerance to gentamicin was restored to above wild type levels for the complemented strain (Table 2). This result could be because the complementation was plasmid-based and consequently done with a high copy number of the *pgmA* gene. All experiments described in the remainder of the results section were done with the defined *S. mutans pgmA* knockout mutant.

**Figure 1:**
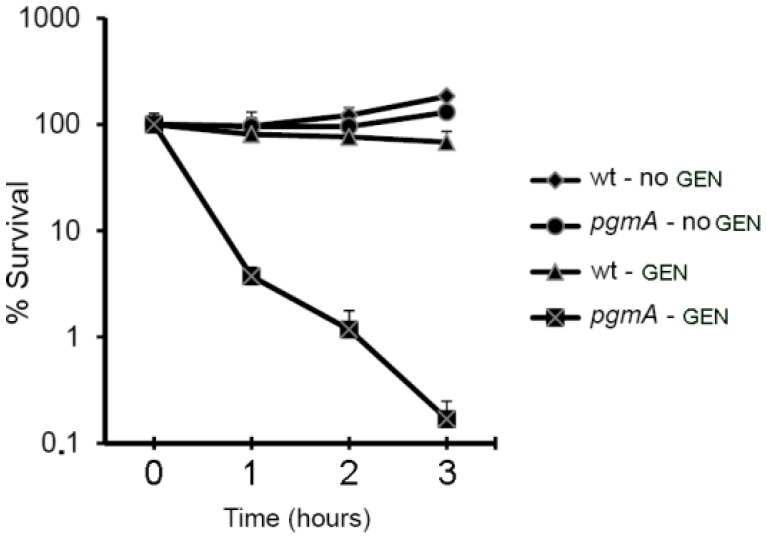
Gentamicin time-kill assay performed on biofilms of the *S. mutans* wild type and *pgmA* mutant. The data are averages of three replicates. Bars indicate standard deviations.

A gentamicin time-kill assay on *S. mutans* wild type and *pgmA* biofilms was conducted to corroborate that *pgmA* mutant biofilms display reduced antibiotic tolerance. Biofilms treated with or without gentamicin were disrupted at various time points by sonication and CFU per biofilm was determined. Gentamicin, at a concentration of approximately half the MBC-B value of the *pgmA* mutant, was used. It was shown to reduce the number of viable *pgmA* mutant cells in the biofilm with 2.8 log within three hours, whereas the same concentration of gentamicin only reduced the number of viable wild-type biofilm cells with 0.2 log within three hours (Figure 1).

Subsequently, we investigated whether the reduced antimicrobial tolerance displayed by *S. mutans pgmA* biofilms transcended to other antibiotics than gentamicin. Biofilms formed by the *S. mutans pgmA* mutant also showed a decrease in MBC-B for vancomycin and linezolid compared to wild-type biofilms (Table 3). Conversely, the vancomycin and linezolid MBC-P values for the *S. mutans pgmA* mutant were only slightly lower than those of the wild-type bacteria (Table 3).

### 3.2. The Amount of Biofilm Formed by the S. mutant pgmA Mutant is Similar to that of the Wild Type

**Figure 2:**
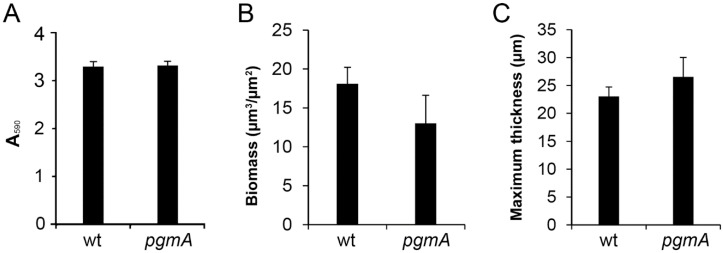
(**A**) Amount of biofilm formed by the *S. mutans* wild type and *pgmA* mutant in microtiter tray wells. Mean and standard deviations of six replicates are shown. (**B**) COMSTAT-calculated biomass of *S. mutans* wild type and *pgmA* mutant biofilms grown on submerged glass surfaces. (**C**) COMSTAT-calculated maximum thickness of *S. mutans* wild type and *pgmA* mutant biofilms grown on submerged glass surfaces. COMSTAT data are represented with mean and standard deviations calculated on six image stacks per experiment.

**Figure 3:**
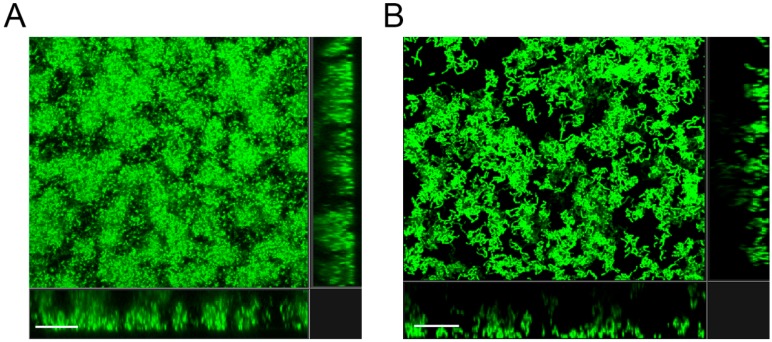
Confocal laser scanning microscopy (CLSM) micrographs of biofilms formed by the *S. mutans* UA159 wild type (**A**) and *pgmA* mutant (**B**) on submerged glass surfaces. The central images show 3-D projections and the flanking images represent vertical sections of the biofilms. Bars correspond to 20 μm.

Because a *Bacillus subtilis pgmA* mutant was reported to be deficient in forming biofilm [29], we investigated whether the lower MBC-B values of the *S. mutans pgmA* mutant could be due to diminished biofilm formation. However, the amount of biofilm formed by the *pgmA* mutant in a microtiter tray-based assay, with conditions as in the MBC-B assays, was not significantly different from the amount of biofilm formed by the wild type (Figure 2A). In addition, CLSM and COMSTAT image analysis on wild-type and *pgmA* mutant biofilms grown on submerged microscope glass slides showed no significant difference in the amount of biofilm biomass and thickness of the biofilms (Figure 2B,C). Moreover, there was no striking difference in the structure of the wild-type and *pgmA* mutant biofilms (Figure 3). These results indicate that the reduced antimicrobial tolerance displayed by the *pgmA* mutant is not caused by a diminished ability to form biofilm.

### 3.3. The S. mutans pgmA Mutant has Altered Cell Wall Properties

**Figure 4:**
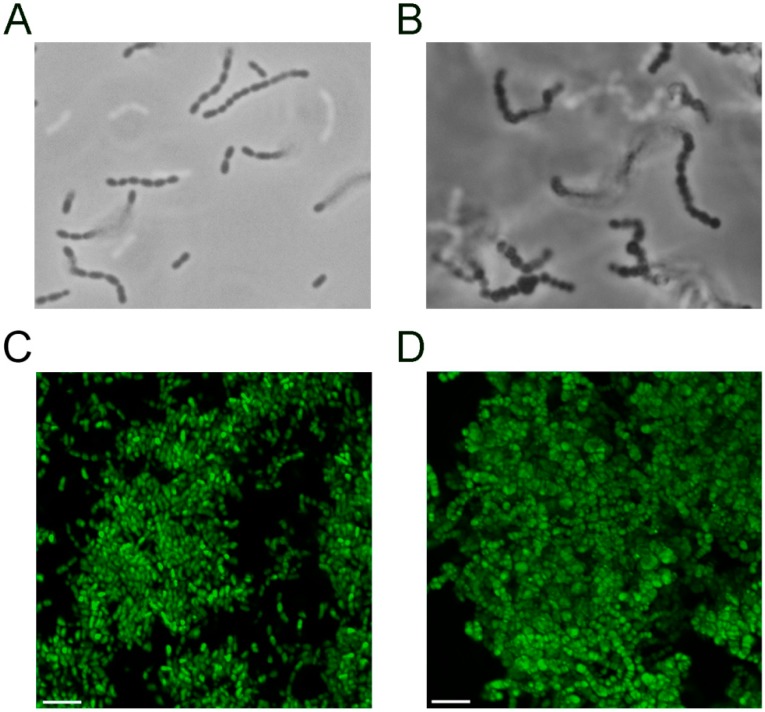
Phase contrast microphotographs (1000 times magnification) of the *S. mutans* UA159 wild type (**A**) and *pgmA* mutant (**B**) from planktonic cultures. Airyscan images of the wild type (**C**) and *pgmA* mutant (**D**) grown as biofilms on submerged glass surfaces. Bars correspond to 4 μm.

Increased cell sizes of *pgmA* mutants of various bacterial species have been reported to be a characteristic feature and to be connected to altered cell wall properties [30,31,32]. Accordingly, microscopy of the *pgmA* mutant and wild type grown in planktonic culture showed that cells of the *pgmA* mutants were larger than wild-type cells (Figure 4A,B). The CLSM micrographs of the wild-type and *pgmA* biofilms (Figure 3) indicated that the *pgmA* mutant also displays increased cell size in biofilms. This was confirmed with Airyscan imaging, which has an increased resolution (Figure 4C,D).

**Figure 5:**
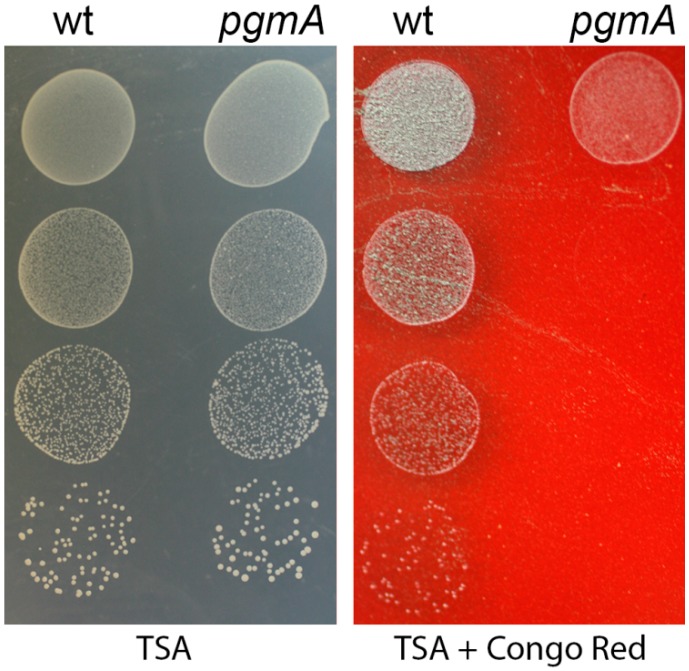
Congo Red susceptibility assay performed on the *S. mutans* wild type and *pgmA* mutant. Ten-fold dilutions of the respective strains were spotted on TSA plates with or without 0.2% Congo red, and the plates were subsequently incubated and imaged.

Reduced cell wall integrity has been shown to correlate with increased sensitivity to the dye Congo Red [33]. To investigate the relative cell wall integrity of the *S. mutans* wild type and *pgmA* mutant, we assayed the sensitivity toward Congo Red. Serial dilutions of the strains were spotted on TSA plates with and without Congo Red. The wild-type strain was not inhibited in colony formation on agar plates with Congo Red, while the *pgmA* mutant showed a marked sensitivity toward Congo Red (Figure 5).

## 4. Discussion

The results presented in this paper firmly establish that inactivation of the *pgmA* gene significantly reduces antimicrobial tolerance of *S. mutans* biofilms, whereas the effect on antibiotic resistance of planktonic bacteria is less pronounced. The PgmA enzyme converts glucose 6-phosphate to glucose 1-phosphate, which is a precursor of UDP-glucose that, in turn, is used in the synthesis of many cell wall components in Gram-positive bacteria, e.g., lipoteichoic acids, wall teichoic acids, membrane glycolipids, capsules, and exopolysaccharides [31]. However, the component(s) whose absence is causing the reduced antibiotic tolerance of *S. mutans pgmA* mutant biofilms have not been identified.

Alanylated lipoteichoic acids have been shown to play a role in the tolerance displayed by streptococcal biofilms to positively charged antibiotics such as aminoglycosides and antimicrobial peptides [16,34], but a role in tolerance to other antibiotics has not been established. Since our *S. mutans pgmA* biofilms showed reduced tolerance to a number of different antibiotics, it is not likely that a lack of lipoteichoic acids is causing the reduced biofilm-associated tolerance displayed by the mutant biofilm.

The matrix of *S. mutans* biofilms consists primarily of glucan exopolysaccharides, extracellular DNA, lipoteichoic acids, and proteins [35]. Our results indicate that the thickness and biomass-content of the *S. mutans* wild type and *pgmA* mutant biofilms did not differ significantly, suggesting that the biofilm matrixes did not differ substantially. It is therefore not likely that the *S. mutans pgmA* mutant and wild type differ substantially in their ability to produce glucan exopolysaccharides of importance for the biofilm structure. Moreover, work done primarily with *P. aeruginosa* has shown that diffusion of antibiotics is generally not inhibited by the biofilm matrix [14]. Limited penetration of antibiotics in biofilms only occurs in cases where the antibiotics bind to components of the extracellular matrix [14]. Minor changes in the biofilm matrix would, therefore, most likely not result in reduced tolerance to several different antibiotics.

We found that the *S. mutans pgmA* mutant cells were larger than wild-type cells. Accordingly, cell size enlargement has been reported for *pgmA* mutants of several different Gram-positive bacteria [30,31,32]. Buchanan et al. demonstrated that a *pgmA* mutant of *Streptococcus iniae* was more sensitive to killing by moronecidin, which is a cationic antimicrobial peptide [32]. They speculated that the increased cell size lowered the rigidity of the cells and made them more prone to lysis caused by antimicrobial peptides acting with pore forming mechanisms. In addition, a lack of glucosylated forms of diacylglycerol, which is correlated with the inability to produce UDP-glucose, has been proposed to play a role in membrane stability, suggesting that *pgmA* mutants have lowered membrane stability [32].

We demonstrated that the *S. mutans pgmA* mutant displayed increased sensitivity to Congo Red, indicating that it has reduced cell wall integrity. Wall teichoic acid has been shown to be of importance for the ability of *Staphylococcus aureus* to grow in the presence of Congo Red [33]. Thus, impaired wall teichoic acid production could be causing the increased Congo Red sensitivity of the *S. mutans pgmA* mutant. Moreover, it is possible that impaired wall teichoic acid production could have a role in the reduced biofilm-associated antibiotic tolerance displayed by the *pgmA* mutant.

*S. mutans pgmA* biofilm cells and planktonic cells displayed similar enlarged cell morphology. This raises the question of why inactivation of the *pgmA* gene significantly reduces antimicrobial tolerance of *S. mutans* biofilms while the effect on antibiotic resistance of planktonic bacteria is less pronounced? One explanation could be that the *pgmA* gene is expressed at a higher level in wild-type biofilm cells compared to planktonic cells, but transcriptomic analysis performed on *S. mutans* biofilm cells and planktonic cells does not support this hypothesis [36]. However, because biofilm cells and planktonically growing cells are physiologically different, membrane integrity could be more important for antibiotic tolerance of biofilm cells than for antibiotic resistance of planktonic cells. Compromised membrane integrity of biofilm cells could result in increased penetration of some types of antibiotics into the cells. A compromised membrane integrity could also lead to higher affinity of antibiotics to the cell wall and increased uptake of antibiotics that require active transport. Biofilm bacteria with compromised membrane integrity could also be more vulnerable to antibiotic-induced stress than planktonic bacteria.

In conclusion, we found that biofilms formed by *S. mutans pgmA* mutant bacteria display reduced tolerance to antibiotics such as gentamicin, vancomycin, and linezolid. The PgmA enzyme is involved in the synthesis of precursors that potentially are of importance for the synthesis of lipoteichoic acids, wall teichoic acids, membrane glycolipids, capsules and exopolysaccharides in *S. mutans*. However, further research is required to identify the component(s) whose absence is causing the reduced antibiotic tolerance of *S. mutans pgmA* mutant biofilms. Increased knowledge about the factors that are involved in biofilm-associated antimicrobial tolerance may ultimately provide a basis for novel strategies for therapeutic interventions against biofilm infections.

## Figures and Tables

**Table 1:** Strains, plasmids and oligonucleotides used in this study.

Strains, Plasmid or Oligonucleotides	Relevant Characteristics or Sequence	Source
***Streptococcus***		
*S. mutans* UA159	American Type Culture Collection (ATCC 700610)	[18]
*S. mutans* UA159 *pgmA*-Tn		This study
*S. mutans* UA159 *pgmA*		This study
*S. mutans* UA159 *pgmA* pDL277		This study
*S. mutans* UA159 *pgmA* pDL277-PgmA		This study
***E. coli***		
HB101	*recA thi pro leu hsdRM*, Sm^r^; strain used for maintainance and proliferation of plasmids	[19]
**Plasmids**		
pDL277	Spec^R^; plasmid used for complementation	[20,21]
**Oligonucleotides**	Sequence	
Erm-PA	GGCGCGCCCCGGGCCCAAAATTTGTTTGAT	
Erm-PB	GGCCGGCCAGTCGGCAGCGACTCATAGAAT	
1077-P1	ACTAGCTTGCGGTGATGTCG	
1077-P2	GGCGCGCCCCTCCTTGGTCTTTTCATCCATTGC	
1077-P3	GGCCGGCCTACTTCAGGTACCGAGCCGAAA	
1077-P4	GTCAGTGAATCTGTTAAAGGTGCT	
1077compF	TATAGGATCCTACGGCGGCAATATCCAGAC	
1077compR	TATAGGATCCTGTTGAACAAGGAAATCATAAAGAC	

**Table 2:** Gentamicin Minimum Bactericidal Concentration for Biofilm Cells (MBC-B) and Minimum Bactericidal Concentration for Planktonic Cells (MBC-P) for transposon, knock-out mutants, and complemented strains (µg/mL). The data are from a representative experiment performed with three replicates. (N.D. means not determined).

	MBC-B	Fold Change	MBC-P	Fold Change
*S. mutans* UA159	300		12	
*S. mutans* UA159 *pgmA*-Tn	38	8×	3	4×
*S. mutans* UA159 *pgmA*	38	8×	3	4×
*S. mutans* UA159 *pgmA* pDL277	38	8×	N.D.	
*S. mutans* UA159 *pgmA* pDL277-PgmA	600	+2×	N.D.	

**Table 3:** Linezolid and vancomycin MBC-B and MBC-P (µg/mL) for the *S. mutans* wild type and derivatives. Fold change indicates the difference in antibiotic concentration compared to wild type. The data are from a representative experiment performed with three replicates. (N.D. means not determined).

	Linezolid MBC-B	Fold Change	Linezolid MBC-P	Fold Change	Vancomycin MBC-B	Fold Change	Vancomycin MBC-P	Fold Change
*S. mutans* UA159	>50		2.5		32		1.25	
*S. mutans* UA159 *pgmA*	12.5	>4×	1.25	2×	8	4x	1.25	1×
*S. mutans* UA159 *pgmA*/pDL277	12.5	>4×	N.D.		8	4x	N.D.	
*S. mutans* UA159 *pgmA*/pDL277-PgmA	>50	1×	N.D.		32	1x	N.D.	
